# Semantic Stability is More Pleasurable in Unstable Episodic Contexts. On the Relevance of Perceptual Challenge in Art Appreciation

**DOI:** 10.3389/fnhum.2016.00043

**Published:** 2016-02-11

**Authors:** Claudia Muth, Marius H. Raab, Claus-Christian Carbon

**Affiliations:** ^1^Department of General Psychology and Methodology, University of BambergBamberg, Germany; ^2^Bamberg Graduate School of Affective and Cognitive SciencesBamberg, Germany; ^3^Forschungsgruppe EPÆG (Ergonomie, Psychologische Æsthetik, Gestaltung)Bamberg, Germany

**Keywords:** Aesthetic Aha, art, dynamic appreciation, semantic instability, predictive coding, episodic context

## Abstract

Research in the field of psychological aesthetics points to the appeal of stimuli which defy easy recognition by being “semantically unstable” but which still allow for creating meaning—in the ongoing process of elaborative perception or as an end product of the entire process. Such effects were reported for hidden images (Muth and Carbon, 2013) as well as Cubist artworks concealing detectable—although fragmented—objects (Muth et al., 2013). To test the stability of the relationship between semantic determinacy and appreciation across different episodic contexts, 30 volunteers evaluated an artistic movie continuously on visual determinacy or liking via the Continuous Evaluation Procedure (CEP, Muth et al., 2015b). The movie consisted of five episodes with emerging Gestalts. In the first between-participants condition, the hidden Gestalts in the movie episodes were of increasing determinacy, in the second condition, the episodes showed decreasing determinacies of hidden Gestalts. In the increasing-determinacy group, visual determinacy was rated higher and showed better predictive quality for liking than in the decreasing-determinacy group. Furthermore, when the movie started with low visual determinacy of hidden Gestalts, unexpectedly strong increases in visual determinacy had a bigger effect on liking than in the condition which allowed for weaker Gestalt recognition after having started with highly determinate Gestalts. The resulting pattern calls for consideration of the episodic context when examining art appreciation.

## Introduction

A specific quality of many experiences with art is the dynamic and inconclusive generation of meaning as can be captured by subjective reports: “[T]he more I looked the more I found, the more I liked and the more I wanted to see more of that work” (interview with an anonymous person; Csikszentmihalyi and Robinson, 1990, p. 57). Research in the field of psychological aesthetics indeed points to the appeal of stimuli which defy an easy recognition but are still evocative of meaning—e.g., hidden images which allow for the rewarding detection of Gestalt (a meaningful cohesive pattern) within seemingly arbitrary patterns (an effect reported as “Aesthetic Aha” by Muth and Carbon, 2013 and replicated for various object categories by Chetverikov and Filippova, 2014). This effect might play an important role in the experience of artworks as well, because they often defy an easy “mastering.” Yet at the same time some artworks enable the active, often inconclusive creation of meaning by the perceiver. For instance, we prefer Cubist artworks which allow for the detection of objects (Kuchinke et al., 2009; Muth et al., 2013) although we are never able to construct a stable determinate Gestalt (Gombrich, 1960/2002). Furthermore, appreciation increased with the detection of Gestalts within artistic movies, and interest went up even prior to these perceptual insights (Muth et al., 2015b). In another study, people generated insights on a variety of semantic levels when elaborating paintings and objects from the nineteenth and twentieth centuries. For instance, they detected Gestalts, interpreted depicted scenes and styles, identified symbols, or reflected on their own perceptual processes. Even though the semantic instability of these artworks was often unresolvable, the experience of such insights had positive effects on interest, liking and powerfulness of affect (Muth et al., 2015a).

Appreciation is repeatedly reported to be positively influenced by the ease of processing (see review by Reber et al., 2004), but the above-mentioned instances are—in contrast— marked by perceptual challenge. We assume here that pleasure is evoked not by easy access to meaning as such, but by the experience of dynamic meaningfulness *within a semantically unstable episodic context*. This specific interplay between semantic stability and unstable episodic contexts might then be qualified by the rewarding experience of insight (Muth and Carbon, 2013). This article consequently presents a study on the relevance of perceptual challenge for the positive effect of insight. It investigates whether a semantically unstable episodic context intensifies the pleasure of creating meaning. Within the scope of this article, we define episodic contexts as qualities of experiences a person gains immediately prior to the actual experience. According to our hypothesis, an experienced perceptual challenge should provide us with the opportunity for a more rewarding insight having a positive effect on liking.

## A dynamic view on semantic stability and appreciation

Artworks are often associated with what we call Semantic Instability (*SeIns* [saIns]; Muth and Carbon, 2015): they challenge easy recognition (like hidden images), allow for a multiplicity of meanings (multistability), promise but defy meaningful patterns (visual indeterminacy) and—at least in the case of representational art—they entail dichotomy between material (e.g., canvas and color), composition and content (e.g., the depicted scene; see Pepperell, 2015). In short: artworks often defy semantic stability by contradicting perceptual habits (Carbon and Leder, 2005; Van de Cruys and Wagemans, 2011). According to fluency theories (e.g., Winkielman et al., 2003) this quality should have a negative effect on appreciation as we supposedly prefer objects that are easy to process. Also Berlyne's (1971) idea of preference for objects of moderate arousal potential with moderate values regarding collative stimulus properties (like complexity, instability, novelty, etc.) contradicts the appeal of *SeIns*: such a high level of arousal potential should cause aversion if we consequently follow this approach. From the perspective of a recently popular approach in the cognitive sciences, Predictive Coding, it is stated that each cognitive system seeks semantic stability: our mental model of the world is continuously actualized to be able to predict the sources of sensual stimulation in the most accurate way (see, e.g., review by Clark, 2013). *SeIns* should therefore cause aversion as situations of low semantic stability are qualified by incongruence between predictions and sensations—so called prediction errors (Van de Cruys and Wagemans, 2011). If we describe these theoretical accounts with a static concept of experience in mind, we might expect a reliable positive link between semantic stability and appreciation because it enables fluency, generates not too much arousal, and creates minimal prediction errors.

**Figure 1:**
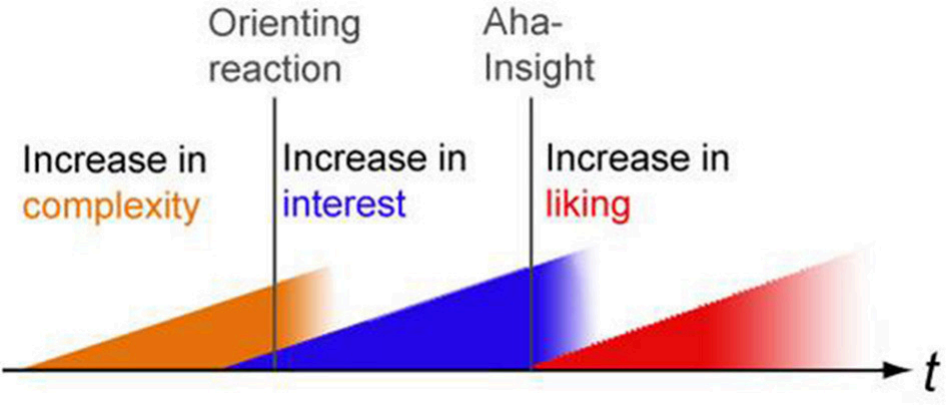
**A preliminary model of dynamics in semantic stability and appreciation; refined version of Figure 13 by Muth et al. (2015b)**.

To investigate the reliability of the relationship between semantic stability and appreciation, we need to make a crucial step beyond this static perspective on perception and appreciation toward a dynamic one (Carbon, 2011; Pelowski and Akiba, 2011). Not only are our perceptual impressions of an object and its context continuously changing due to changes in position of our body and the world, meaning evolves out of our interaction with the world and is not inherent to it. Semantic stability is never preset but a result of this interaction (see Muth and Carbon, 2015; Muth et al., 2015b). In addition to these physical and semantic changes, we need to consider their dynamic linkage with appreciation: e.g., repeated presentation of initially semantically unstable objects might cause increased familiarity, and thus increases positive affect (mere-exposure effect, Zajonc, 1968; limited by boredom, see Bornstein, 1989; or fatigue, Carbon, 2011). Not only does un-reinforced exposure influence appreciation: it could be shown that innovative designs are liked more with increased depth of elaboration (engagement with various qualities of the design; see Repeated Evaluation Technique, RET; Carbon and Leder, 2005). And the formation of semantic stability within hidden images yields a sudden increase in liking (Muth and Carbon, 2013). There are at least four interconnected lines of theoretical argumentation able to explain such dynamics concerning the relationship between semantic stability and appreciation:

- As much as *SeIns* is not a static property of an object, the fluency of its processing is subject to changes, too. We can expect it to change with exposure (see mere-exposure effect as explained above) or elaboration (see repeated-evaluation effects as explained above). And furthermore, insight experiences might be accompanied by a rewarding, sudden and strong increase in processing fluency (as proposed, e.g., by Topolinski and Reber, 2010).
- Berlyne (1971) suggested the existence of two reward systems: one would be linked to the moderate increase in arousal potential and deactivated by values too low or too high, as described above. The other one would be linked to decreases in arousal potential until a moderate level is reached. An initially high level of arousal through *SeIns* might therefore decrease during elaboration to arrive at such a pleasurable level.
- Van de Cruys and Wagemans (2011) developed an idea based on Predictive Coding which is capable of explaining how initially aversive reactions to prediction errors induced by an artwork might be transformed into positive affect: predictive progress, the reduction of a mismatch between predictions about the cause of the experience and the actual experience, might itself be rewarding.
- In the case of artworks, such a decrease in *SeIns* might not always be progressive or finite. It might even have a negative effect on appreciation if ambiguity is too easily resolvable (Hyman, 2010; Muth et al., 2015a). Instead, it might be more fruitful to focus on dynamic transformational processes in the perceiver after the encounter of *SeIns* involving insight and self-change rather than the rather blunt pleasure of a mastery (Pelowski and Akiba, 2011). Muth and Carbon (2015) suggested that insights during the elaboration of an artwork might temporarily increase positive affect, maybe even several times during the perception of one and the same artwork and even without a complete resolution of *SeIns.* This is for instance the case if we get a clue about a fragmented object concealed in a Cubist artwork by identifying the strings of a violin, but lose it again in the next moment when focusing on contradictory elements within the painting. Furthermore, even the mere anticipation of meaningful insights already showed an increase of interest (Muth et al., 2015b). Interest is indeed frequently reported to link “disorientation” with a “promise of success” (Berlyne, 1971) or challenge with coping potential (Silvia, 2005), respectively. Muth et al. (2015b) proposed a preliminary model (see Figure 1) integrating these findings: an increase in complexity causes an orienting reaction due to expected meaningfulness, and with that evokes interest as a kind of “affective forecasting” (Wilson and Gilbert, 2003) of a rewarding insight. If an insight occurs, liking increases (“Aesthetic Aha” effect).

The approaches sketched here are partially linked as they all associate the formation of meaning with pleasure. One point that needs differentiated consideration however is the role of perceptual challenge (or “disruption” see Pelowski and Akiba, 2011 with reference to Dewey): the concept of pleasure by processing fluency is linked to a variety of object features like prototypicality and symmetry (e.g., Reber et al., 2004) without the need for a challenging episodic context. Berlyne's (1971) idea of reward by decrease in arousal, in contrast, presupposes a precedent high level of arousal. It was indeed suggested that the mere-exposure effect—which can be understood as a process of arousal reduction by reduction of novelty—is most relevant to complex stimuli as their difficulty prevents an early influence of boredom: “Presumably, simple stimuli become boring more quickly than complex stimuli, resulting in a more rapid downturn in the frequency-affect curve” (Bornstein, 1989, p. 279). Also Van de Cruys and Wagemans (2011) explicitly claim that pleasure might benefit from an encounter with a prediction error as it enables the rewarding predictive progress in the first place. Indeed, in a study by Dörner and Vehrs (1975) patterns were most appreciated when they concealed an order that is not recognizable initially, but only after effort.

To address the relevance of effort in the link between semantic stability and appreciation, we need to take the episodic context into consideration and thus find a way to qualify dynamics in the experience of art. In the study to be presented we asked how reliable the positive link between semantic stability and appreciation is, concretely: is the link stronger in semantically unstable episodic contexts, when an event was preceded by a stronger perceptual challenge?

## Materials and methods

### Participants

Participants were grouped in two sets: increasing-determinacy group (15 participants, 14 females; mean_age_ = 20.6 years; range_age_ = 18–27 years) and decreasing-determinacy group (15 participants, 13 females; mean_age_ = 22.9 years; range_age_ = 19–29 years). A *Snellen* eye chart test and a test with a subset of the *Ishihara* color cards assured that all of them had normal or corrected-to-normal visual acuity and normal color vision. The participants were naïve to the purpose of the study.

### Apparatus and stimuli

**Figure 2:**
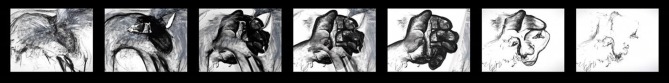
**Exemplary frames of the stop-motion movie “Konstrukte” by Claudia Muth (from the year 2009)**. Image courtesy of Claudia Muth (see Supplementary Material for the two versions of the full movie).

**Figure 3:**
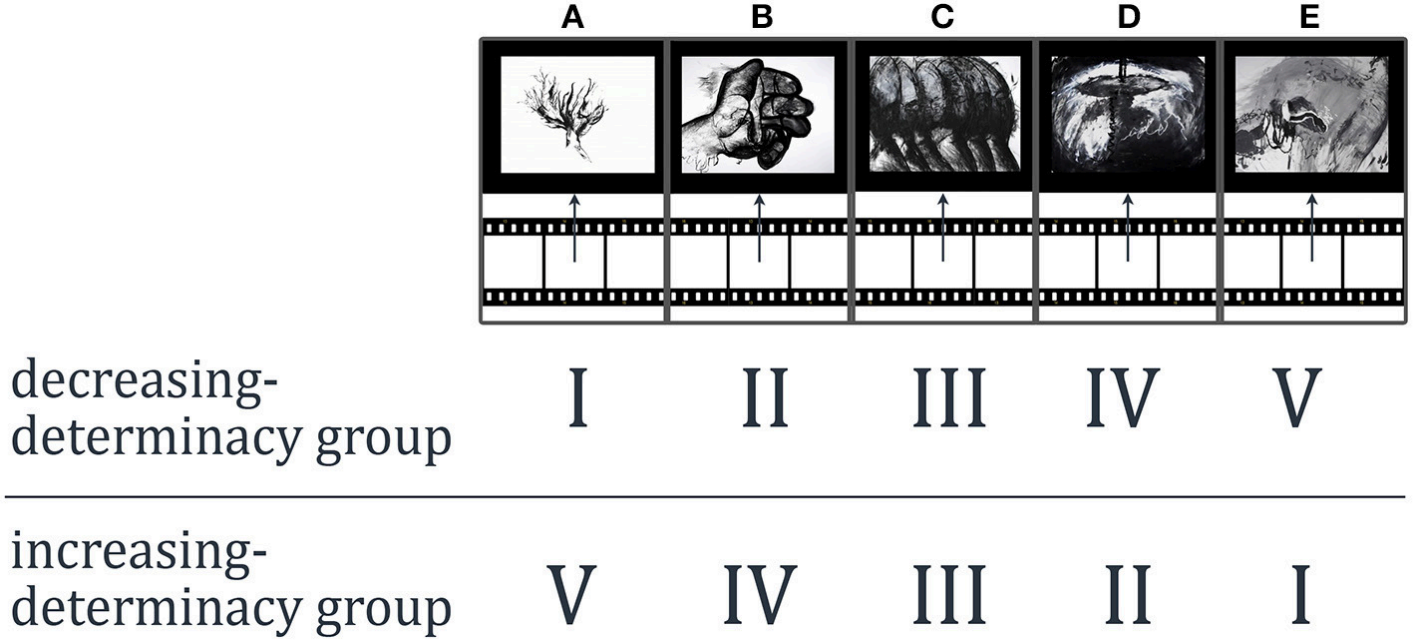
**Order of the episodes A–E for each between-participants condition and one movie frame of each episode exemplifying the differences in determinacy of Gestalt**.

As stimulus material we utilized a movie consisting of *five* episodes which dynamically reveal and conceal Gestalts (see Figure 2 and Supplementary Material for *two* versions of the movie). “Konstrukte” (07:18 min.) by Claudia Muth (from the year 2009) was originally not created as experimental material but has been used as such before by Muth et al. (2015b). The artistic movie documents an intuitive drawing technique (charcoal and acrylic paint) by fusing thousands of photographs at different stages of the drawing process (stop-motion technique). Gestalt evolves out of arbitrarily drawn lines and painted blots; order slowly evolves and Gestalt morphs into new Gestalt or dissolves. Being a dynamic stimulus, it allows for investigating the effects of dynamic variations of *SeIns* and includes the evocation of predictions as well as prediction errors due to the associations and expectations of the perceiver. We used *two* different versions of the movie: *one* in which *five* episodes were combined in an order of increasing visual determinacy of detectable Gestalts (episode order: A–B–C–D–E), with the *first* movie-episode A being the least evocative of clear Gestalt and the last movie-episode E being most evocative of clear Gestalt (watched by the increasing-determinacy group). A *second* version showed the same episodes in an order of decreasing visual determinacy (episode order: E–D–C–B–A; watched by the decreasing-determinacy group, see Figure 3 for a visualization of these orders and Supplementary Material for full movies). Note that the order of episodes was reversed, not the order of the whole movie based on an inversed frame number. Furthermore, the episodes were combined in such a way that they appeared as *one* cohesive movie (as provided in the Supplementary Material) without any apparent break between episodes. Participants were also not alerted to the fact that the movie was broken into episodes for the experimental procedure. The *five* episodes were of different length. The movie was presented on a LG W2220P screen with a 22-inch screen size and a resolution of 1680 × 1050 pixels.

As an assessment method we utilized the Continuous Evaluation Procedure (CEP; developed by the research network Ergonomics, Psychological Æsthetics and Gestaltung, *EPÆG*; Muth et al., 2015b). CEP uses a slider box as the input device which captures assessments in a very time-accurate way (standard lever; 100 mm movement range, 10 kΩ linear characteristics mounted on wooden housing). An ATMEGA microprocessor maps the lever's resistance to a value between 80 (lowest) and 1024 (highest lever position). We will refer to this resistance as “strength” in the following and use a transposed scale ranging from 0 to 1000. The value is transferred to the connected PC via an FTDI serial-to-USB converter and the current slider position is updated constantly by the ATMEGA processor and requested via the Serial-to-USB interface for each frame of the movie (30 frames per second in this case). We implemented the video presentation via the Processing Library for Visual Arts and Design (Fry and Reas, 2014) and the GStreamer library (Open-Source, 2014).

### Procedure

**Figure 4:**
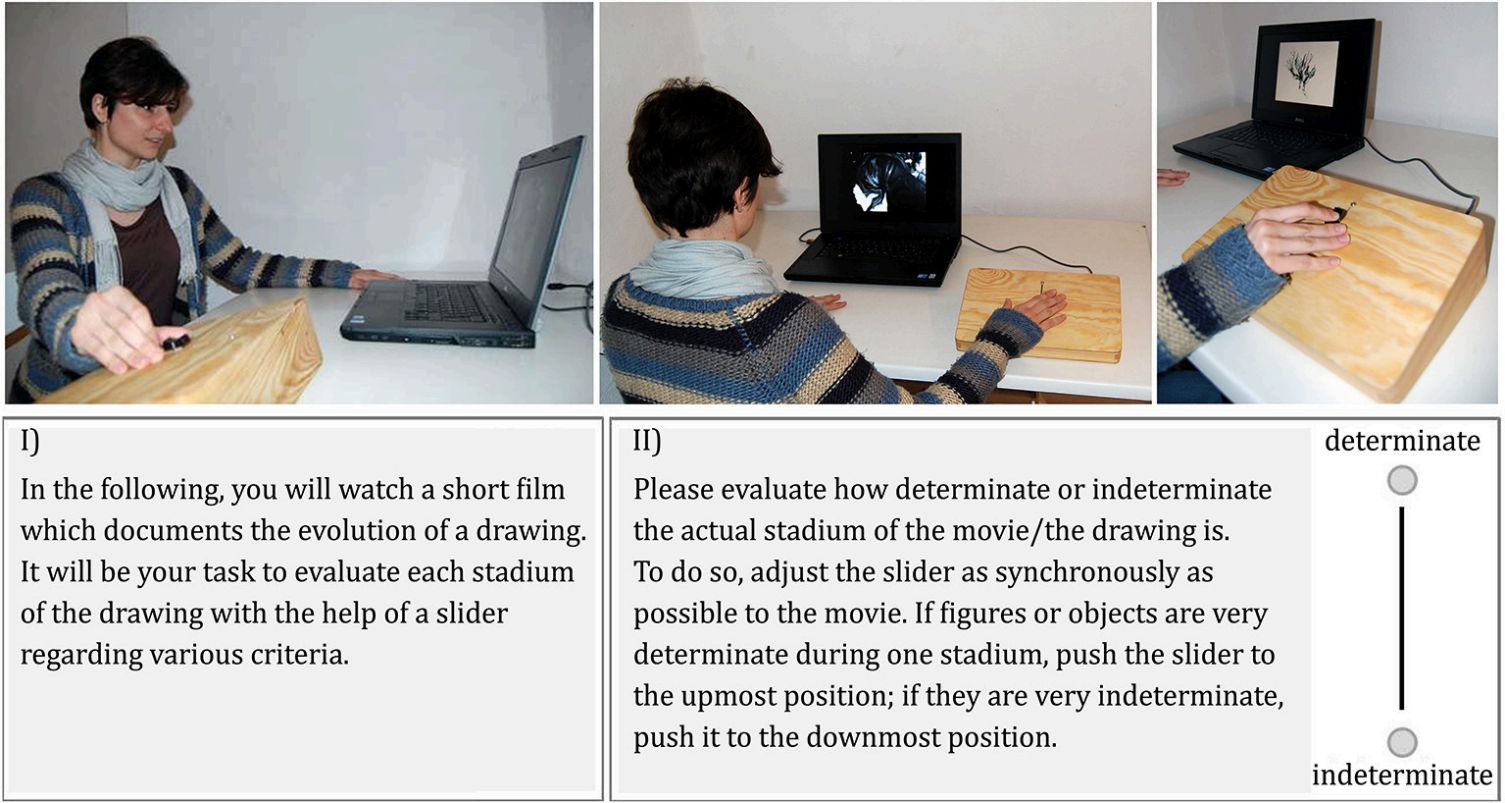
**(I) Introduction to the study as given to the participants and (II) exemplary instruction for the key variable “determinacy” (translated from German)**.

Participants watched the artistic movie twice. Each time they evaluated it continuously on one dimension via the CEP: each person evaluated it first on determinacy (as a measure of semantic stability) and in a second trial on liking (as a measure of appreciation) or vice versa (counterbalanced design). The increasing-determinacy group watched the *five* episodes in an order of increasing determinacy with the first movie-episode being the least evocative of clear Gestalt and the last movie-episode being the most evocative of clear Gestalt. In a second between-participants condition, participants of the decreasing-determinacy group watched these episodes vice-versa in an order of decreasing determinacy (see Figure 3). The exact directions given to participants are reported in Figure 4. These instructions were added by a graphical representation of the slider and the two poles of the according key dimension for better understanding (see Figure 4). Before the movie started, participants were asked to get familiar with the usability of the slider by moving it up and down.

### Ethical statement

Before the experiment, participants gave written informed consent to participating in the study. After the experiment had ended participants were fully informed about the aims of the study and had the opportunity to ask questions. All data were collected anonymously and no harming procedures were used. The reported study was approved by the “Ethikrat der Universität Bamberg” (ethics committee of the University of Bamberg).

## Results

### Reliability of the relationship across different episodic contexts

Each movie-frame was represented by an average value of strength of determinacy and of liking for each between-participant-condition. In order to exclude any possible bias with regard to the comparison of the between-participant-conditions we first checked for order-effects on the evaluation of determinacy or liking that might have been caused by the counter-balanced design. This analysis showed no significant differences (Δ for liking = 16.92 with higher ratings when liking was evaluated during the second block; Δ for determinacy = 11.05 with higher ratings when determinacy was evaluated during the second block). Furthermore, an ANOVA with episodic context and order as between-participant-factors did not produce any significant interaction.

**Figure 5:**
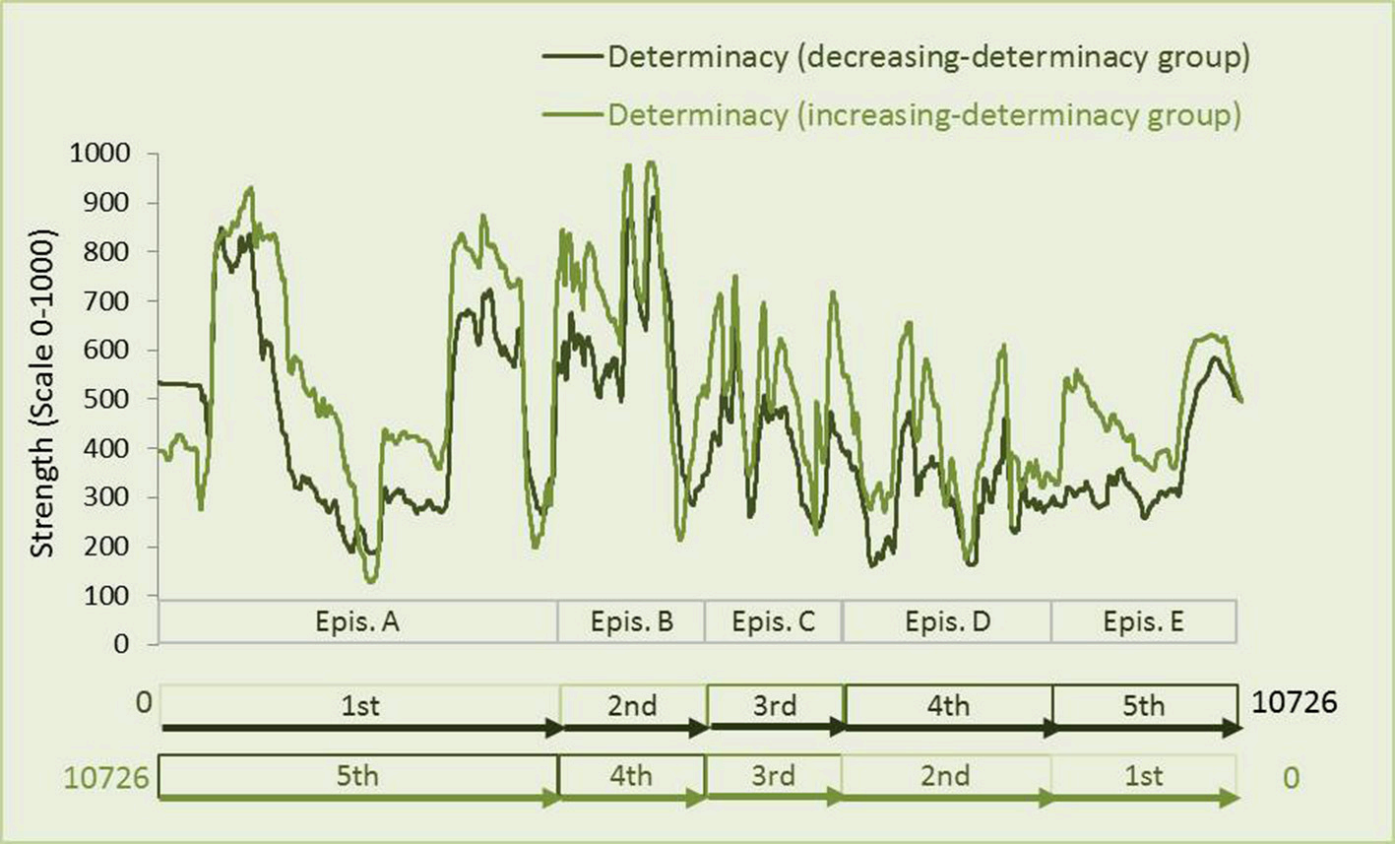
**Differences in strength of visual determinacy between the increasing-determinacy group and the decreasing-determinacy group**. The Figure shows evaluations of the decreasing-determinacy group in the original order and evaluations of the increasing-determinacy group in a rearranged fashion so that evaluations of episode A now appear to the left although they were given at the end. Note the inversed order of frames for the data assessed by the increasing-determinacy group: the movie was not shown inversed based on frame number but based on the order of five episodes (A–E). Numbers indicate the order of episodes.

**Figure 6:**
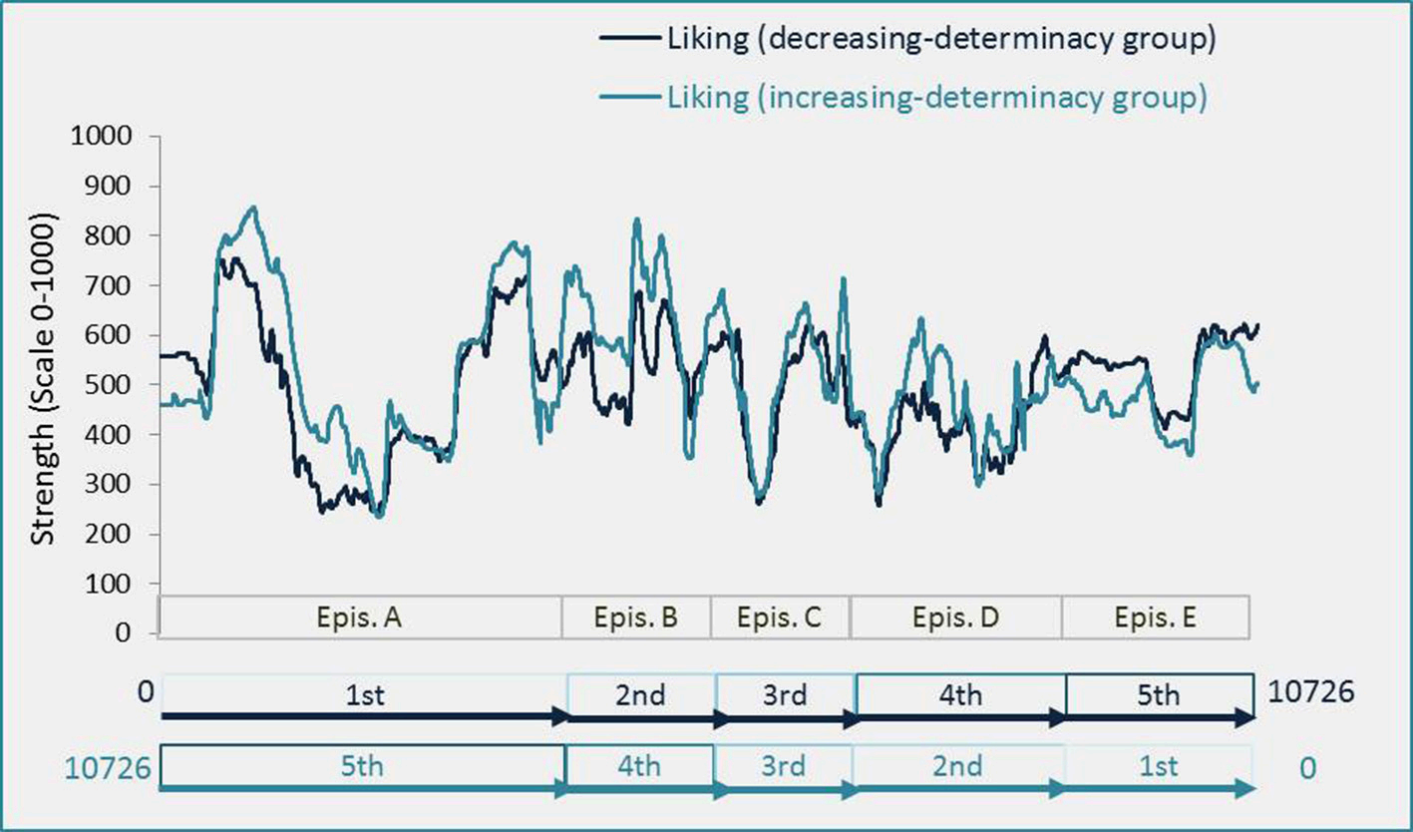
**Differences in strength of liking between the increasing-determinacy group and the decreasing-determinacy group**. The Figure shows evaluations of the decreasing-determinacy group in the original order and evaluations of the increasing-determinacy group in a rearranged fashion so that evaluations of episode A now appear to the left although they were given at the end. Note the inversed order of frames for the data assessed by the increasing-determinacy group: the movie was not shown inversed based on frame number but based on the order of five episodes (A–E). Numbers indicate the order of episodes. Note the inversed order of frames for the data assessed by the increasing-determinacy group: the movie was not shown inversed based on frame number, but on the order of five episodes (A–E).

**Figure 7:**
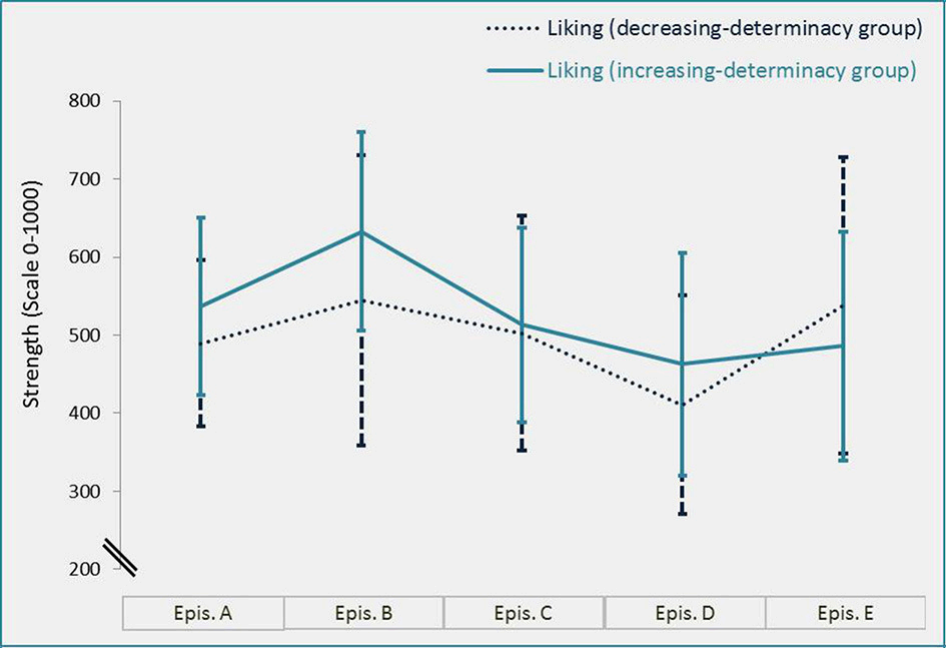
**Differences in liking between episodes and episodic context**. Error bars represent ±1 *SD*.

A paired *t*-test revealed that in the increasing-determinacy group, determinacy [*M* = 98.22, *t*_(10725)_ = 102.53, *p* < 0.001; *d* = 0.99; see Figure 5] and liking [*M* = 27.78, *t*_(10725)_ = 34.47, *p* < 0.001, *d* = 0.33; see Figure 6] were generally rated higher than in the decreasing-determinacy group. We conducted an additional mixed Analysis of Variance (ANOVA) with data being aggregated over frames per participant and episode to take a closer look at the evaluation of each episode on liking in the two episodic contexts. It included the repeated-measures factor of episode (1–5) and the between-participants factor of episodic context (increasing-determinacy vs. decreasing-determinacy) and liking as dependent variable. Although liking evaluations were higher for episodes A–D in the increasing-determinacy group than in the decreasing-determinacy group (see Figure 7), the interaction between episode and episodic context did not reach significance [*F*_(4, 112)_ = 2.213, *p* = 0.072]. It can therefore only be speculated rather than stated with confidence that in the increasing-determinacy group, episodes A–D benefited from the precedent experience of *SeIns* in episode E being the first episode in the increasing-determinacy group and the last one in the decreasing-determinacy group.

**Figure 8:**
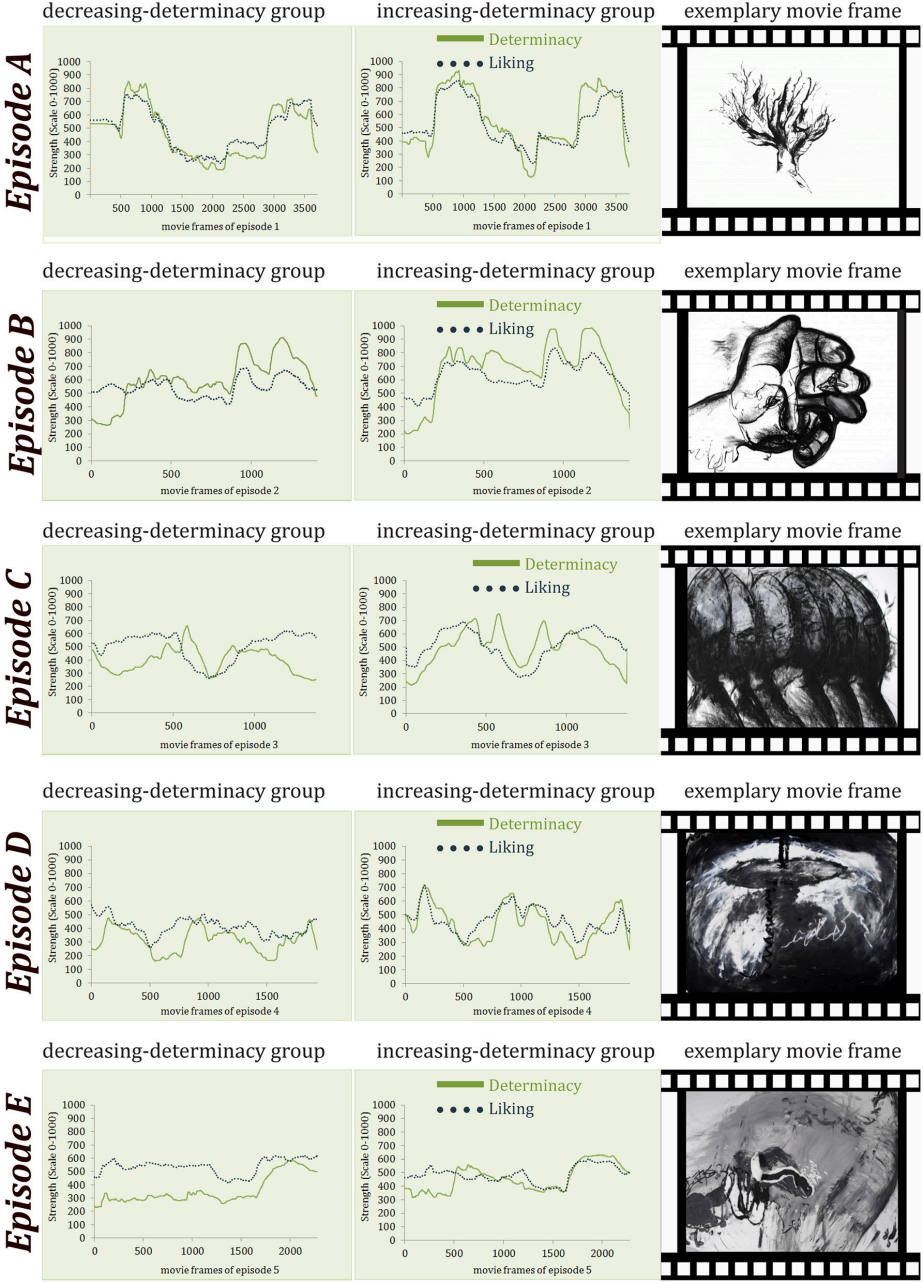
**Determinacy and liking evaluations over time (movie frames) from the decreasing-determinacy group and the increasing-determinacy group**. Note that episodes differ in length (frame number) as they were chosen based on content and not originally created as stimulus material.

Furthermore, a Pearson correlation revealed that visual determinacy showed better predictive quality for liking in the increasing-determinacy group (*R*^2^ = 0.71, *t*_(10724)_ = 163.16, *p* < 0.001) than in the decreasing-determinacy group (*R*^2^ = 0.47, *t*_(10724)_ = 101.83, *p* < 0.001; see Figure 8).

### Reliability of the temporary increase in liking at moments of insight across different episodic contexts

To furthermore assess the temporary effects of a perceptual insight during Gestalt recognition on liking (“Aesthetic Aha”), we compared increases in liking at moments of insight between the two conditions. Muth et al. (2015b) already identified seven moments of insight from the same movie as was utilized in the current study. These were defined as peaks in evaluations of determinacy and surprise collected in the study by Muth et al. (2015b). We compared liking evaluations before and after these seven moments of insight between the two conditions to reveal if insights had a greater effect in the increasing-determinacy than in the decreasing-determinacy group.

**Figure 9:**
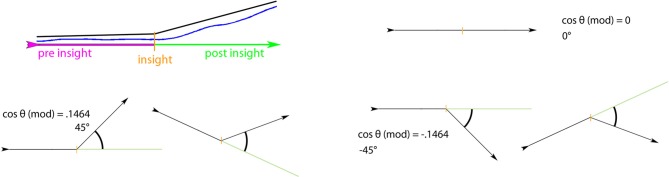
**The modified cosine theta measure to capture dissimilarity between pre- and post-insight**. The data section before the insight as well as after the insight was approximated with a line each (top left). Between these two vectors, the angle was determined. Two vectors with exactly the same direction, i.e., with no difference in direction pre- and post-insight, result in an angle of 0° between vectors and thus in a cosine theta measure of 0. A directional change upwards (second vector pointing higher than the first one) will result in a positive theta (e.g., 0.1464 for a 45° upward angle between vectors), a directional change downward in a negative theta value (e.g., −0.1464 for a 45° downward angle).

To quantify the change in a given dimension for a given point in time, we used a modified cosine value, based on the angle between the line describing data before (frame “-60” to frame “0”) and the line describing data after the moment in question (frame “0” to frame “60”; see Figure 9). The cosine measure is a common means for evaluating the similarity between two arbitrary, n-dimensional vectors (see Singhal, 2001). As we wanted to capture the *dissimilarity* between two vectors (i.e., the difference between the trends before and after insight), we have modified the cosine formula. Now it results in “0” when both vectors (pre and post the moment to be evaluated) have the same direction; it approaches “1” when the post-vector marks an increase compared to the pre-vector (where “0.1464” would be an angle of 45° and “0.5” would be an angle of 90°); and it approaches “−1” when the post-vector marks a decrease (see Figure 9).

While the usual advantage of the cosine measure is its ability to handle *n-dimensional* vectors, we have also chosen to use this (slightly modified) approach for our two-dimensional purpose. The cosine measure lends itself to the comparison of angles (and thus, differences) between vectors. It emphasizes large differences/angles in contrast to small differences (due to the cosine distribution); and it is, at the same time, similar to a Pearson correlation with values bounded between −1 and 1 (Egghe and Leydesdorff, 2009).

**Figure 10:**
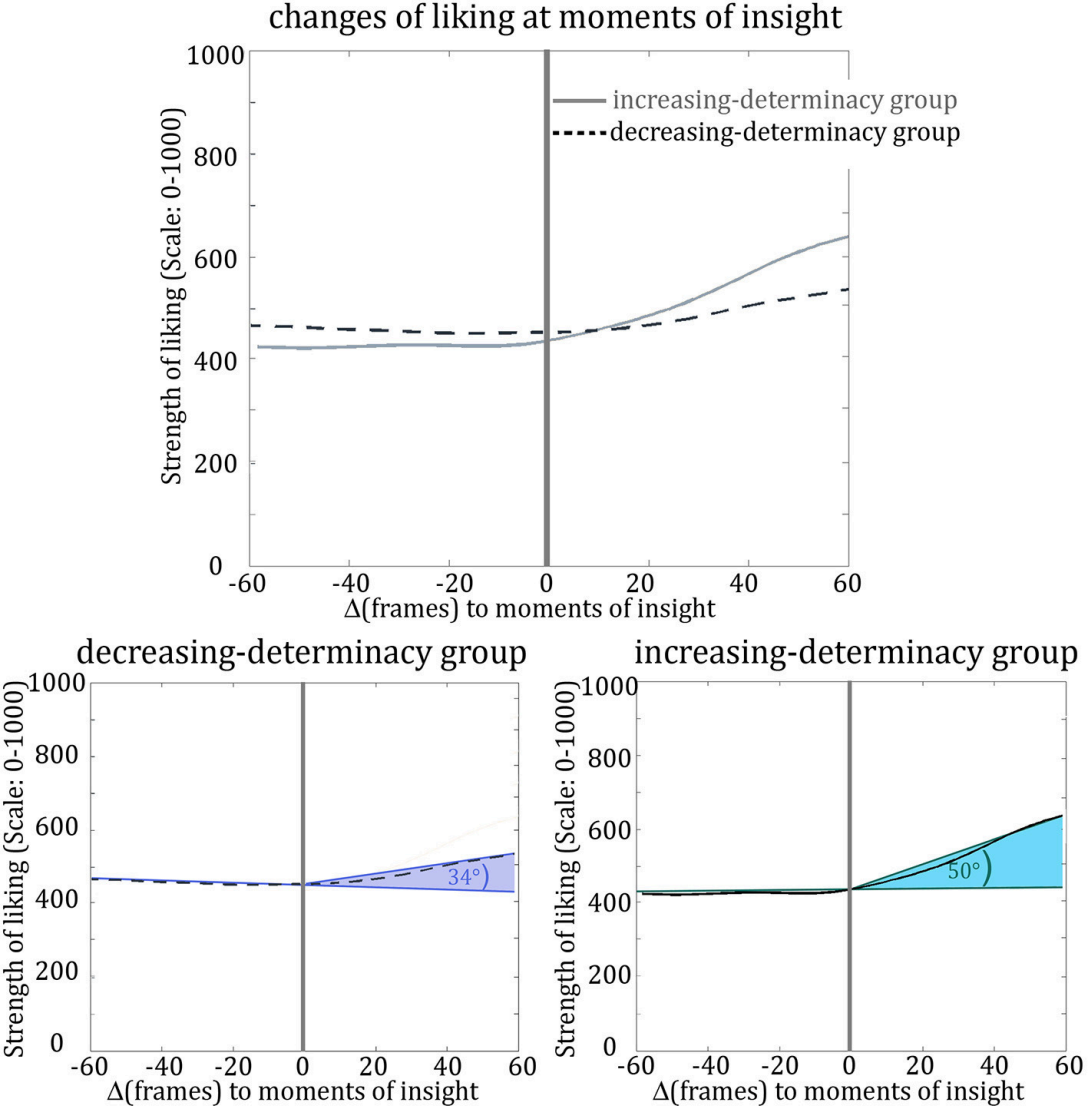
**Dynamics of liking in relation to moments of insight for the increasing-determinacy group and the decreasing-determinacy group**. “0” marks liking at the moments of insight, “60” marks liking at 60 frames after moments of insight, “−60” marks liking 60 frames before moments of insight, etc.

Figure 10 shows the average change in liking at moments of insight. To see if this change differs significantly from other changes in liking during the movie's elaboration, we compared the modified cosine values extracted for each of the seven moments of insight to those of 1000 randomly picked data intervals. We conducted a two-sided independent *t*-test in MATLAB (version R2011b, The MathWorks, Inc.; via the function “ttest2” which accounts for unequal sample sizes using a Welch correction). This analysis revealed that for each between-participant condition, the change in liking at the seven moments of insight is different from general, random changes. For the increasing-determinacy group the modified cosine value was 0.179 and the according angle 50.059° [*t*_(1005)_ = 2.476, *p* = 0.013, Cohen's *d* = 1.504], for the decreasing-determinacy group the modified cosine value was 0.087 and the according angle 34.310° [*t*_(1005)_ = −2.687, *p* = 0.007, Cohen's *d* = 0.344; see Figure 10]. The increase in liking at moments of insight was not only greater for the increasing-determinacy group on average, but also for each of the seven insights except one (the changes do not reach significance on every single moment of insight though, see Table 1).

**Table 1:** **Angles between the slope describing liking from 60 frames before an insight to liking at the moment of insight and the slope connecting liking from the moment of insight to 60 frames after it for each between-participants group**.

**Episode**	**Moments of insight (movie frame)**	**Decreasing-determinacy group angle (mod. cosine)**	**Increasing-determinacy group angle (mod. cosine)**	**Δ Increasing-/Decreasing-determinacy group Δ(angle)**
A	490	37.81° (0.105)	53.84°(0.205)^*^	16.03°
A	2183	24.22° (0.044)	49.91° (0.178)	25.69°
A	2863	38.74° (0.110)	41.06° (0.123)	2.32°
B	3868	−20.93° (−0.033)	24.50° (0.045)	45.43°
B	3900	0° (0)	45.89° (0.152)	45.89°
B	4576	73.74° (0.360)	72.30° (0.348)^*^	−1.44°
D	6577	−48.02° (−0.166)^*^	58.53° (0.239)^*^	106.55°

Significant changes in liking (p ≤ 0.05) are marked by an asterisk.

## Discussion

We investigated the relevance of perceptual challenge to the positive effect of semantic stability on appreciation and asked whether a semantically unstable episodic context intensifies the pleasure of creating meaning. To do so we manipulated the order of movie episodes differing in the degree of *SeIns* (Semantic Instability, see Muth and Carbon, 2015). Evaluations of persons who were initially exposed with higher *SeIns* (i.e., increasing-determinacy group) were higher regarding liking as well as visual determinacy and revealed a stronger link between visual determinacy and liking. Furthermore, an analysis of the dynamic link between insights and liking revealed a stronger “Aesthetic Aha” effect (increase in liking at moments of insight, Muth and Carbon, 2013) for persons who watched the episodes in an order of increasing visual determinacy. It can be concluded that not only might semantic stability as such be appreciated, but even more so increases in semantic stability after the encounter of perceptual challenge (e.g., Dörner and Vehrs, 1975; Van de Cruys and Wagemans, 2011; Muth and Carbon, 2015). Thus, episodic contexts in which we experience great difficulty in deciphering Gestalt allow for more rewarding increases in certainty than less visually indeterminate contexts. Consequently, when the movie started with more visually indeterminate phases, an increase in visual determinacy had a bigger effect on liking. The resulting pattern calls for considering episodic context when investigating phenomena of *SeIns*. Furthermore, our findings underline that, while according to Predictive Coding accounts we aim at an increase in predictive accuracy or a reduction of surprise respectively (see also the principle of minimizing free energy by Friston, 2005), the pleasure of predictive progress might be greater in unstable semantic contexts. Seen within a bigger picture, this is one possible explanation for the fact that we do not avoid stimulation to keep prediction errors low (an issue discussed as the “dark room problem,” Friston et al., 2012): people challenge their perceptual habits, for instance, by visiting art exhibitions, by exploring new countries, by listening to music that plays with transformations and contradictions to expected motives (Weth et al., 2015), or by learning difficult games. They might do so at least partially because the gain of insights in challenging situations induces pleasure—presumably even more so than the mere encounter of familiar (fluent) situations. This effect might be additionally influenced by the “safety” of the overall situational frame (e.g., museum, non-threatening situations at holiday or leisure time; see Carbon et al., 2013 for effects of feeling safe on ratings of innovativeness). The feeling of safety might as well influence the motivational context: we can assume that people go into a *preservation mode* if safety is low (goals associated with higher security and familiarity, linked to “prettiness”) and a *promotion mode* if safety is high (goals associated with mental growth and novelty, linked to “beauty,” see for a differentiation of these motivational modes; Armstrong and Detweiler-Bedell, 2008):

When promotion focused, a person will seek novelty to gain new cognitive structures for coping with the world. Alternatively, when prevention focused, a person will avoid ambiguity and inconsistency to prevent confusion and to maintain existing knowledge structures and belief systems.

(Armstrong and Detweiler-Bedell, 2008, p. 319).

A promotion mode might be beneficial for the pleasure of forming meaning within *SeIns*. It even seems to be a precondition for engaging with challenging situations in the first place. In contrast, fluently processed objects might be preferred if a person is in a prevention mode (as suggested by Armstrong and Detweiler-Bedell, 2008).

As much as it is an oversimplification to describe the perception and appreciation of art via stable evaluations, it would be an oversimplification to describe the effect of artworks on the perceiver via two dimensions of semantic stability and liking. First, it is evident that “semantic stability”—or, in more general terms, “meaningfulness”—comprises a much larger set of phenomena than those of visual determinacy. Second, affective reactions might range from mild pleasure to being moved, physiological reactions of chills and thrills and even an extraordinary and sometimes life-changing experience of Aesthetic “Awe” and the sublime (Konečni, 2010). Pelowski and Akiba (2011), for instance, pointed to the crucial relevance of disruption and transformation in art perception which seem to be neglected widely in current accounts in psychological aesthetics in favor of examining the hedonic value of harmonious, fluent experiences of meaning. Although still ill-defined up to this point, several psychological accounts describe *the* “dependent variable” of aesthetic perception with much more detail than is applied in many empirical studies in the field. Armstrong and Detweiler-Bedell (2008), for instance, analyze the concept of “free beauty” with reference to Kant as a blend between non-fluency and the promise of meaningfulness (associated with a promotion focus, see above) and differentiate it from “prettiness,” mere liking or a mundane pleasure which would be rather closely linked to fluency effects (associated with a prevention focus, see above). Indeed, variables like *SeIns* might affect mere liking to a different extent than alternative variables like interest or powerfulness of affect (see, e.g., Ishai et al., 2007; Muth et al., 2015a). Furthermore, not every artwork might cause (or even try to cause) pleasure or feelings of beauty—even if insights generated during its elaboration might be judged valuable or interesting (for an important differentiation between positive effects on interest and negative ones on pleasure by disturbing art see Turner and Silvia, 2006). On the other hand, the positive effect of instable episodic contexts might be especially relevant in art perception: it would be interesting to investigate if participants would have benefited from a semantically instable context in the same way if they would not have classified the movie as an art work. Additionally, the valence of generated insights (e.g., a positive or negative expression of a hidden face) could influence appreciation (see Fluency Amplification Model by Albrecht and Carbon, 2014). It is therefore crucial to extend our findings of the influence of semantically unstable episodic contexts on liking to additional varieties of meaningfulness and affect.

We showed that the link between *SeIns* and liking is influenced by episodic context: perceptual challenge amplified the positive effect of visual determinacy on liking. In subsequent attempts at investigation it has to be further revealed which art-specific varieties of semantic stability exist beyond mere visual determinacy, and how according processes are related to several experiential qualities during art perception.

## Author contributions

CM developed the main idea for the study and refined its detailed procedure together with CC and MR. The apparatus was developed by MR, supported by CM and CC, testing was covered by CM, MR, and CC advised and conducted part of the analysis.

### Conflict of interest statement

The authors declare that the research was conducted in the absence of any commercial or financial relationships that could be construed as a potential conflict of interest.

## References

[B1] Albrecht S. Carbon C. C. (2014). The fluency amplification model: fluent stimuli show more intense but not evidently more positive evaluations. Acta Psychol. 148, 195–203. 10.1016/j.actpsy.2014.02.002 24603044

[B2] Armstrong T. Detweiler-Bedell B. (2008). Beauty as an emotion: the exhilarating prospect of mastering a challenging world. Rev. Gen. Psychol. 12, 305–329. 10.1037/a0012558

[B3] Berlyne D. E. (1971). Aesthetics and Psychobiology. New York, NY: Appleton-Century-Crofts.

[B4] Bornstein R. F. (1989). Exposure and affect: overview and meta-analysis of research, 1968–1987. Psychol. Bull. 106, 265–289. 10.1037/0033-2909.106.2.265

[B5] Carbon C. C. (2011). Cognitive mechanisms for explaining dynamics of aesthetic appreciation. Iperception 2, 708–719. 10.1068/i0463aap 23145254 PMC3485809

[B6] Carbon C. C. Faerber S. J. Gerger G. Forster M. Leder H. (2013). Innovation is appreciated when we feel safe: on the situational dependence of the appreciation of innovation. Int. J. Design 7, 43–51.

[B7] Carbon C. C. Leder H. (2005). The repeated evaluation technique (RET): a method to capture dynamic effects of innovativeness and attractiveness. Appl. Cogn. Psychol. 19, 587–601. 10.1002/acp.1098

[B8] Chetverikov A. Filippova M. (2014). How to tell a wife from a hat: affective feedback in perceptual categorization. Acta Psychol. (Amst). 151, 206–213. 10.1016/j.actpsy.2014.06.012 25051145

[B9] Clark A. (2013). Whatever next? Predictive brains, situated agents, and the future of cognitive science. Behav. Brain Sci. 36, 181–204. 10.1017/S0140525X12000477 23663408

[B10] Csikszentmihalyi M. Robinson R. E. (1990). The Art of Seeing: An Interpretation of the Aesthetic Encounter. Los Angeles, CA: J. Paul Getty Museum and the Getty Education Institute for the Arts.

[B11] Dörner D. Vehrs W. (1975). Aesthetical appreciation and reduction of uncertainty. Psychol. Res. Psychol. Forschung 37, 321–334. 10.1007/BF00309726

[B12] Egghe L. Leydesdorff L. (2009). The relation between Pearson's correlation coefficient *r* and Salton's cosine measure. J. Am. Soc. Inf. Sci. 60, 1027–1036. 10.1002/asi.21009

[B13] Friston K. (2005). A theory of cortical responses. Philos. Trans. R. Soc. 360, 815–836. 10.1098/rstb.2005.1622 15937014 PMC1569488

[B14] Friston K. Thornton C. Clark A. (2012). Free-energy minimization and the dark room problem. Front. Psychol. 3:130. 10.3389/fpsyg.2012.00130 22586414 PMC3347222

[B15] Fry B. Reas C. (2014). Processing Library for Visual Arts and Design. Available online at: http://www.processing.org

[B16] Gombrich E. H. (1960/2002). Art Illusion: A Study in the Psychology of Pictorial Representation, 5th Edn. Oxford: Phaidon Press.

[B17] Hyman J. (2010). Art and neuroscience, in Beyond Mimesis and Convention, eds Frigg R. Hunter M. (Dordrecht; Heidelberg; London; New York, NY: Springer), 245–261.

[B18] Ishai A. Fairhall S. L. Pepperell R. (2007). Perception, memory and aesthetics of indeterminate art. Brain Res. Bull. 73, 319–324. 10.1016/j.brainresbull.2007.04.009 17562398

[B19] Konečni V. I. (2010). Aesthetic trinity theory and the sublime. Proc. Eur. Soc. Aesthetics 2, 244–264. 10.5840/philtoday201155162

[B20] Kuchinke L. Trapp S. Jacobs A. M. Leder H. (2009). Pupillary responses in art appreciation: effects of aesthetic emotions. Psychol. Aesthet. Creativity Arts 3, 156–163. 10.1037/a0014464

[B21] Muth C. Carbon C. C. (2013). The Aesthetic Aha: on the pleasure of having insights into Gestalt. Acta Psychol. (Amst). 144, 25–30. 10.1016/j.actpsy.2013.05.001 23743342

[B22] Muth C. Carbon C. C. (2015). SeIns. Semantic instability in art. Art Percept. 4, 145–184. 10.1163/22134913-00002049

[B23] Muth C. Hesslinger V. Carbon C. C. (2015a). The appeal of challenge in the perception of art: how ambiguity, solvability of ambiguity and the opportunity for insight affect appreciation. Psychol. Aesthet. Creativity Arts 9, 206–216. 10.1037/a0038814

[B24] Muth C. Pepperell R. Carbon C. C. (2013). Give me Gestalt! Preference for cubist artworks revealing high detectability of objects. Leonardo 46, 488–489. 10.1162/LEON_a_00649

[B25] Muth C. Raab M. Carbon C. C. (2015b). The stream of experience when watching artistic movies. Dynamic aesthetic effects revealed by the continuous evaluation procedure (CEP). Front. Psychol. 6:365. 10.3389/fpsyg.2015.00365 25873907 PMC4379740

[B26] Open-Source (2014). GStreamer. Available online at: http://gstreamer.freedesktop.org/

[B27] Pelowski M. Akiba F. (2011). A model of art perception, evaluation and emotion in transformative aesthetic experience. New Ideas Psychol. 29, 80–97. 10.1016/j.newideapsych.2010.04.001

[B28] Pepperell R. (2015). Artworks as dichotomous objects: implications for the scientific study of aesthetic experience. Front. Hum. Neurosci. 9:295. 10.3389/fnhum.2015.00295 26106312 PMC4460548

[B29] Reber R. Schwarz N. Winkielman P. (2004). Processing fluency and aesthetic pleasure: is beauty in the perceiver's processing experience? Pers. Soc. Psychol. Rev. 8, 364–382. 10.1207/s15327957pspr0804_3 15582859

[B30] Silvia P. J. (2005). Cognitive appraisals and interest in visual art: exploring an appraisal theory of aesthetic emotions. Empir. Stud. Arts 23, 119–133. 10.2190/12AV-AH2P-MCEH-289

[B31] Singhal A. (2001). Modern information retrieval: a brief overview. Bull. IEEE Comput. Soc. Tech. Comm. Data Eng. 24, 35–42.

[B32] Topolinski S. Reber R. (2010). Gaining insight into the “Aha” experience. Curr. Dir. Psychol. Sci. 19, 402–405. 10.1177/0963721410388803

[B33] Turner S. A. Jr. Silvia P. J. (2006). Must interesting things be pleasant? A test of competing appraisal structures. Emotion 6, 670–674. 10.1037/1528-3542.6.4.670 17144758

[B34] Van de Cruys S. Wagemans J. (2011). Putting reward in art: a tentative prediction error account of visual art. Iperception 2, 1035–1062. 10.1068/i0466aap 23145260 PMC3485793

[B35] Weth K. Raab M. H. Carbon C. C. (2015). Investigating emotional responses to self-selected sad music via self-report and automated facial analysis. Musicae Sci. 19 412–432. 10.1177/1029864915606796

[B36] Wilson T. D. Gilbert D. T. (2003). Affective forecasting. Adv. Exp. Soc. Psychol. 35, 345–411. 10.1016/S0065-2601(03)01006-2

[B37] Winkielman P. Schwarz N. Fazendeiro T. Reber R. (2003). The hedonic marking of processing fluency: implications for evaluative judgment, in The Psychology of Evaluation: Affective Processes in Cognition and Emotion, eds Musch J. Klauer K. C. (Hillsdale, MI: Lawrence Erlbaum Associates), 189–217.

[B38] Zajonc R. B. (1968). Attitudinal effects of mere-exposure. J. Pers. Soc. Psychol. 9, 1–27. 10.1037/h00258485667435

